# Cardiomyocyte adhesion and hyperadhesion differentially require ERK1/2 and plakoglobin

**DOI:** 10.1172/jci.insight.140066

**Published:** 2020-09-17

**Authors:** Maria Shoykhet, Sebastian Trenz, Ellen Kempf, Tatjana Williams, Brenda Gerull, Camilla Schinner, Sunil Yeruva, Jens Waschke

**Affiliations:** 1 Faculty of Medicine, Ludwig-Maximilians-University Munich, Munich, Germany.; 2 Comprehensive Heart Failure Center and Department of Internal Medicine I, University Hospital Würzburg, Würzburg, Germany.

**Keywords:** Cardiology, Arrhythmias, Cardiovascular disease, Cell migration/adhesion

## Abstract

Arrhythmogenic cardiomyopathy (AC) is a heart disease often caused by mutations in genes coding for desmosomal proteins, including desmoglein-2 (DSG2), plakoglobin (PG), and desmoplakin (DP). Therapy is based on symptoms and limiting arrhythmia, because the mechanisms by which desmosomal components control cardiomyocyte function are largely unknown. A new paradigm could be to stabilize desmosomal cardiomyocyte adhesion and hyperadhesion, which renders desmosomal adhesion independent from Ca^2+^. Here, we further characterized the mechanisms behind enhanced cardiomyocyte adhesion and hyperadhesion. Dissociation assays performed in HL-1 cells and murine ventricular cardiac slice cultures allowed us to define a set of signaling pathways regulating cardiomyocyte adhesion under basal and hyperadhesive conditions. Adrenergic signaling, activation of PKC, and inhibition of p38MAPK enhanced cardiomyocyte adhesion, referred to as positive adhesiotropy, and induced hyperadhesion. Activation of ERK1/2 paralleled positive adhesiotropy, whereas adrenergic signaling induced PG phosphorylation at S665 under both basal and hyperadhesive conditions. Adrenergic signaling and p38MAPK inhibition recruited DSG2 to cell junctions. In PG-deficient mice with an AC phenotype, only PKC activation and p38MAPK inhibition enhanced cardiomyocyte adhesion. Our results demonstrate that cardiomyocyte adhesion can be stabilized by different signaling mechanisms, which are in part offset in PG-deficient AC.

## Abstract 2

Desmosome mediated cardiomyocyte adhesion, crucial in the pathology of arrhythmogenic cardiomyopathy, is differentially regulated by multiple signaling mechanisms that depend either on ERK1/2 or plakoglobin.

## Introduction

In the heart, cardiomyocytes are coupled via intercalated disks (ICD) consisting of desmosomes, adherens junctions (AJ), and gap junctions, which together allow mechanical stability and electrical conduction of the heart muscle (1). Cardiomyocytes express desmosomal cadherins desmoglein-2 (DSG2) and desmocollin-2 (DSC2) and the AJ protein N-cadherin (N-CAD) (2). The cytosolic part of these adhesion molecules connects to the cytoskeletal network via the plaque proteins plakoglobin (PG), plakophilin (PKP), and desmoplakin (DP) in desmosomes or to β-catenin and other plaque proteins in AJs, respectively. Adjacent cardiomyocytes attach via DSG2, DSC2, and N-CAD, which bind to their respective partners extracellularly in a Ca^2+^-dependent manner (3). Cardiomyocyte function markedly relies on the proper function of desmosomal and AJ proteins, because mutations in these genes lead to an arrhythmic heart disorder called arrhythmogenic cardiomyopathy (AC) (4, 5). The progressive loss of cardiomyocytes with cardiac fibrosis, ventricular dilatation, and arrhythmia, accompanied by a high risk for sudden cardiac death, especially in young athletes, are the characteristics of AC. The mechanisms by which desmosomal components serve heart function and how cardiomyocyte adhesion is regulated in pathological conditions are poorly understood. Thus, therapy for AC is symptom based and focused on limiting arrhythmia in patients. In this context, desmosomal components are thought to regulate conduction of excitation via gap junction and the cardiac sodium channel complex allowing ephaptic conduction (6, 7). Therefore, characterization of mechanisms regulating desmosomal adhesion can help to establish new experimental approaches to treat desmosomal diseases, as have been shown for pemphigus (1), a disease caused by autoantibodies primarily against DSG1 and DSG3, leading to severe blistering of the skin and mucosa (8, 9). For pemphigus, the mechanisms controlling keratinocyte adhesion have been studied extensively and were found to focus on p38MAPK, PKC, ERK1/2, and cAMP signaling (10–12), whereas others, such as SRC, appear not to be central for the disease (13). The context of pemphigus is relevant to AC for 2 reasons: First, AC is known to occur in syndromes that affect the skin and its appendices when specific mutations in genes coding for PG or DP are causative (14–16). Second, recently, it has been proposed that different mutations in desmosomal genes causing AC can induce the formation of autoantibodies against cardiomyocyte antigens, including DSG2, which are pathogenic and thus may aggravate the disease (17, 18). This would imply that the mechanisms causing AC and pemphigus may be at least in part comparable. Therefore, in order to establish new therapeutic approaches for AC, it is pivotal to characterize the molecular mechanisms regulating cardiomyocyte adhesion in detail. Such therapeutic approaches might focus on enhancing cardiomyocyte adhesion and thus prevent fibro-fatty replacement with the subsequent occurrence of arrhythmias.

Although desmosomal cadherins depend on the presence of extracellular Ca^2+^, it was shown that in keratinocytes desmosomes acquire a Ca^2+^-independent state, referred to as hyperadhesion, which was proposed to be the primary adhesive state of desmosomes in intact epidermis (19–21). This phenomenon was not explored further in other tissues. Indeed, in a previous study, we found that in cardiomyocytes adrenergic signaling leads to the enhancement of basal cellular adhesion, which we referred to as positive adhesiotropy, and also causes hyperadhesion (22). Since cardiac muscle contractions depend on Ca^2+^, which is tightly regulated intra- and extracellularly, it is possible that a hyperadhesive state similar to keratinocytes exists in the physiologic state of desmosomes in cardiomyocytes. Thus, the signaling mechanisms regulating hyperadhesion in cardiomyocytes need to be investigated.

In multiple heart diseases, including myocardial dysfunction, diabetic cardiomyopathy, hypertrophy, fibrosis, and heart failure, MAPK signaling pathways, such as ERK1/2, JNK, and p38MAPK, are demonstrated to be involved in disease pathogenesis (23–25). Besides, PKC has been shown to be important in the regulation of gap junctions in cardiomyocytes (26, 27). Moreover, in previous studies, we found that activation of PKC (28) and adrenergic signaling enhance cardiomyocyte adhesion, the latter of which is mediated through PKA pathway–induced PG phosphorylation at S665 (22).

In this study, we investigated the signaling pathways that enhance cardiomyocyte adhesion and may induce hyperadhesion of desmosomal contacts. Our results show that enhancement of cAMP levels by adrenergic signaling, activation of PKC, as well as inhibition of p38MAPK enhance basal cardiomyocyte adhesion and cause hyperadhesion. ERK1/2 was found to be critical to enhance basal cardiomyocyte adhesion, i.e., for positive adhesiotropy, which was dependent on PG, DP, and DSG2 in cultured cardiomyocytes. In ventricular cardiac slices obtained from PG-deficient mice with an AC phenotype, adrenergic signaling failed to enhance cardiomyocyte adhesion, whereas PKC activation and p38MAPK inhibition were still effective. On the other hand, ERK1/2 activation did not play a role in hyperadhesion caused by the above signaling pathways, indicating that the mechanisms to enhance cardiomyocyte adhesion, at least in part, are different between basal and hyperadhesive conditions.

## Results

### Adrenergic signaling, PKC, and p38MAPK regulate cardiomyocyte adhesion in HL-1 cells and murine ventricular cardiac slices.

**Figure 1:**
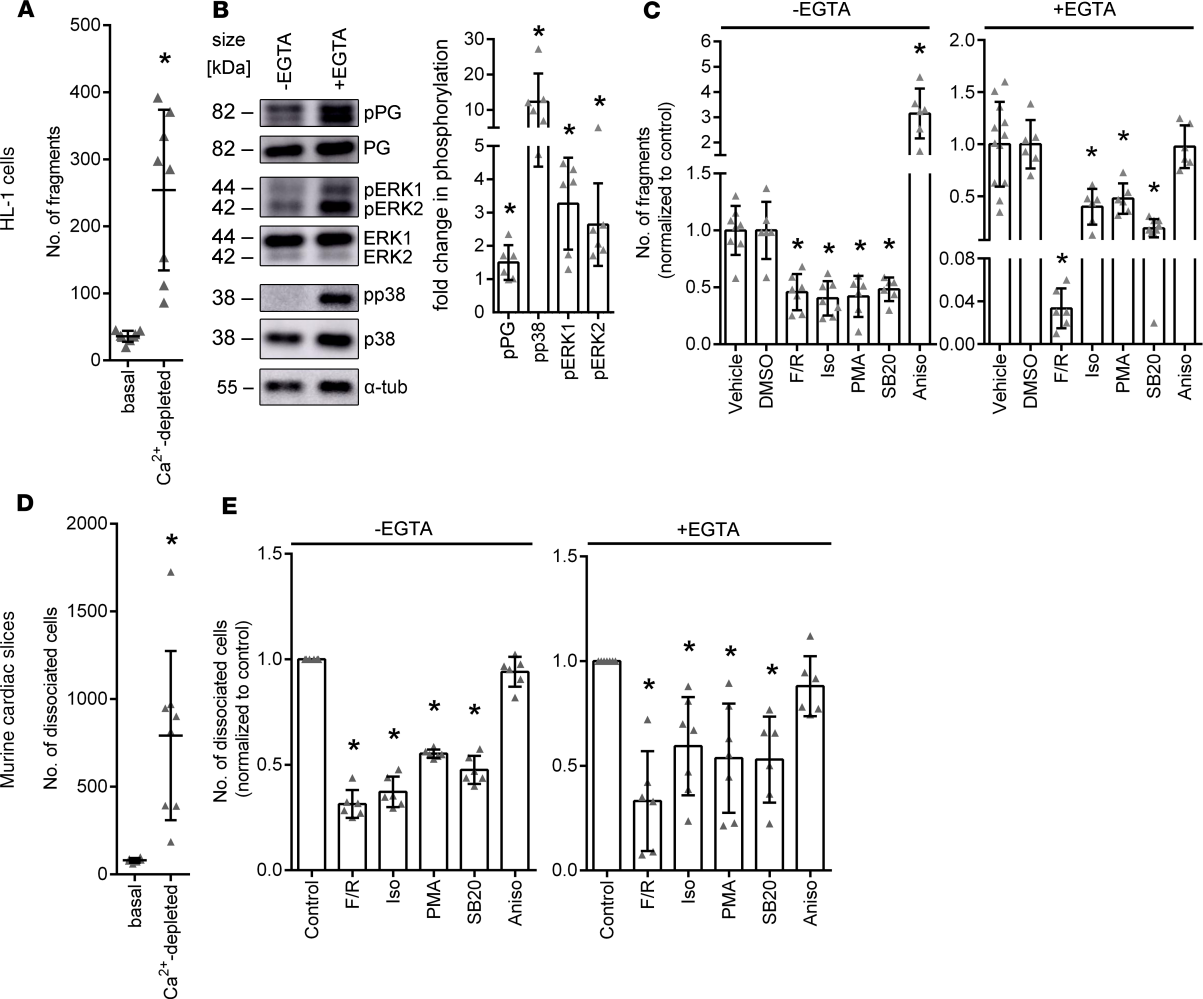
Cardiomyocyte cohesion regulation by different signaling pathways. (**A**) Dissociation assays in HL-1 cardiomyocytes under basal and Ca^2+^-depleted conditions. *P ≤ 0.05, unpaired Student’s *t* test, *n* = 8. (**B**) Representative Western blots showing changes in signaling pathways between basal and Ca^2+^-depleted conditions. Fold changes in phosphorylation compared with –EGTA (basal conditions) are indicated. *P ≤ 0.05, unpaired Student’s *t* test, *n* = 6. (**C**) Dissociation assays in HL-1 cells, showing fold change of the number of fragments after treatment with F/R, Iso, PMA, SB20, or Aniso under basal (–EGTA) or Ca^2+^-depleted (+EGTA) conditions as compared with respective controls. DMSO serves as control for SB20. *P ≤ 0.05, 1-way ANOVA with Holm-Šidák correction, *n* = 6. (**D**) Dissociation assays in cardiac slice cultures obtained from WT mice in basal and Ca^2+^-depleted conditions. *P ≤ 0.05, unpaired Student’s t test, *n* = 8. (**E**) Dissociation assays in cardiac slice cultures obtained from WT mice, showing fold change of the number of dissociated cells after treating with F/R, Iso, SB20, PMA, or Aniso under basal and Ca^2+^-depleted conditions, normalized to the respective control slices. *P ≤ 0.05, 1-way ANOVA with Holm-Šidák correction, *n* = 6.

In keratinocytes, a Ca^2+^-independent desmosomal adhesion, which is called hyperadhesion, has been identified (19–21). In a previous study, we observed a similar phenomenon in cultured HL-1 cardiomyocytes (22). In this study, we first determined the Ca^2+^ dependency of cardiomyocyte adhesion using dissociation assays. In dissociation assays, cells are treated with dispase and collagenase to detach cell monolayers from the well bottoms, and a standardized mechanical stress is applied to the monolayers. The resulting fragmentation serves as an indirect measurement of cell-cell cohesion; a higher amount of fragmentation indicates a weaker cell-cell cohesion and vice versa. However, it has to be noted that the assay measures overall cell cohesion and cannot discriminate between adhesion provided by desmosomal cadherins and classical cadherins, such as N-CAD. Dissociation assays showed that upon Ca^2+^ depletion cardiomyocyte adhesion reduced strongly, with the number of fragments increasing from 41.41 ± 18.38 fragments in basal conditions to 233.32 ± 102.73 fragments in Ca^2+^-depleted conditions (Figure 1A). This was paralleled by a significant increase in phosphorylation of PG, p38MAPK, and ERK1/2 (Figure 1B), suggesting that these signaling events might be involved in the regulation of intercellular adhesion of HL-1 cells. To investigate the role of PKC, p38MAPK, ERK1/2, and adrenergic signaling in basal and Ca^2+^-depleted conditions further, we performed dissociation assays in HL-1 cells after treating the cells with respective mediators. We used forskolin and rolipram (F/R, a combination of an adenylyl cyclase activator and a phosphodiesterase-4 inhibitor), isoprenaline (Iso, a β-adrenergic receptor agonist), phorbol-12-myristate-13-acetate (PMA, a PKC activator), SB202190 (SB20, a p38MAPK-inhibitor), and anisomycin (Aniso, a p38MAPK activator) similar to previous studies on desmosomal adhesion in cardiomyocytes and keratinocytes (22, 28–33). We also used the β-adrenergic receptor blockers propranolol (Pro) and bisoprolol (Bis) due to their implications in AC disease treatment. We did not find any changes in cardiomyocyte cohesion upon treatment with β-adrenergic receptor blockers under basal conditions. (Supplemental Figure 2; supplemental material available online with this article; https://doi.org/10.1172/jci.insight.140066DS1). We observed that under basal conditions, adrenergic signaling, PKC activation, and p38MAPK inhibition enhanced HL-1 cell adhesion, whereas activation of p38MAPK markedly reduced cell adhesion. Similarly, under Ca^2+^-depleted conditions, adrenergic signaling, PKC activation, and p38MAPK inhibition led to a hyperadhesive state of cardiomyocyte adhesion, as revealed by a significant reduction of fragment numbers (Figure 1C). In contrast, activation of p38MAPK did not further reduce cellular adhesion upon Ca^2+^ depletion.

Next, we evaluated whether cardiomyocyte adhesion is regulated by comparable mechanisms in murine ventricular cardiac slice cultures using a modified dissociation assay. Ca^2+^ depletion strongly decreased cardiomyocyte adhesion in cardiac slice cultures, as seen in HL-1 cells, with the amount of dissociated cells increasing to 791.47 ± 451.17 in Ca^2+^-depleted conditions from 80.09 ± 11.89 dissociated cells in basal conditions (Figure 1D). Similar to experiments in HL-1 cells, adrenergic signaling, PKC activation, and p38MAPK inhibition enhanced cardiomyocyte adhesion under basal conditions and induced hyperadhesion (Figure 1E) upon Ca^2+^ depletion. p38MAPK activation did not affect cell adhesion in cardiac slice cultures, neither under basal nor Ca^2+^-depleted conditions. The experiments demonstrate that in HL-1 cardiomyocytes the principle mechanisms regulating cardiomyocyte adhesion are comparable to the ones in ventricular cardiomyocytes of intact heart tissue. Moreover, the data show that adrenergic signaling, PKC activation, and p38MAPK inhibition increase cellular adhesion and can induce hyperadhesion in cardiomyocytes.

### Adrenergic and PKC signaling differentially regulate PG phosphorylation as well as ERK1/2 and p38MAPK activation in HL-1 cells.

**Figure 2:**
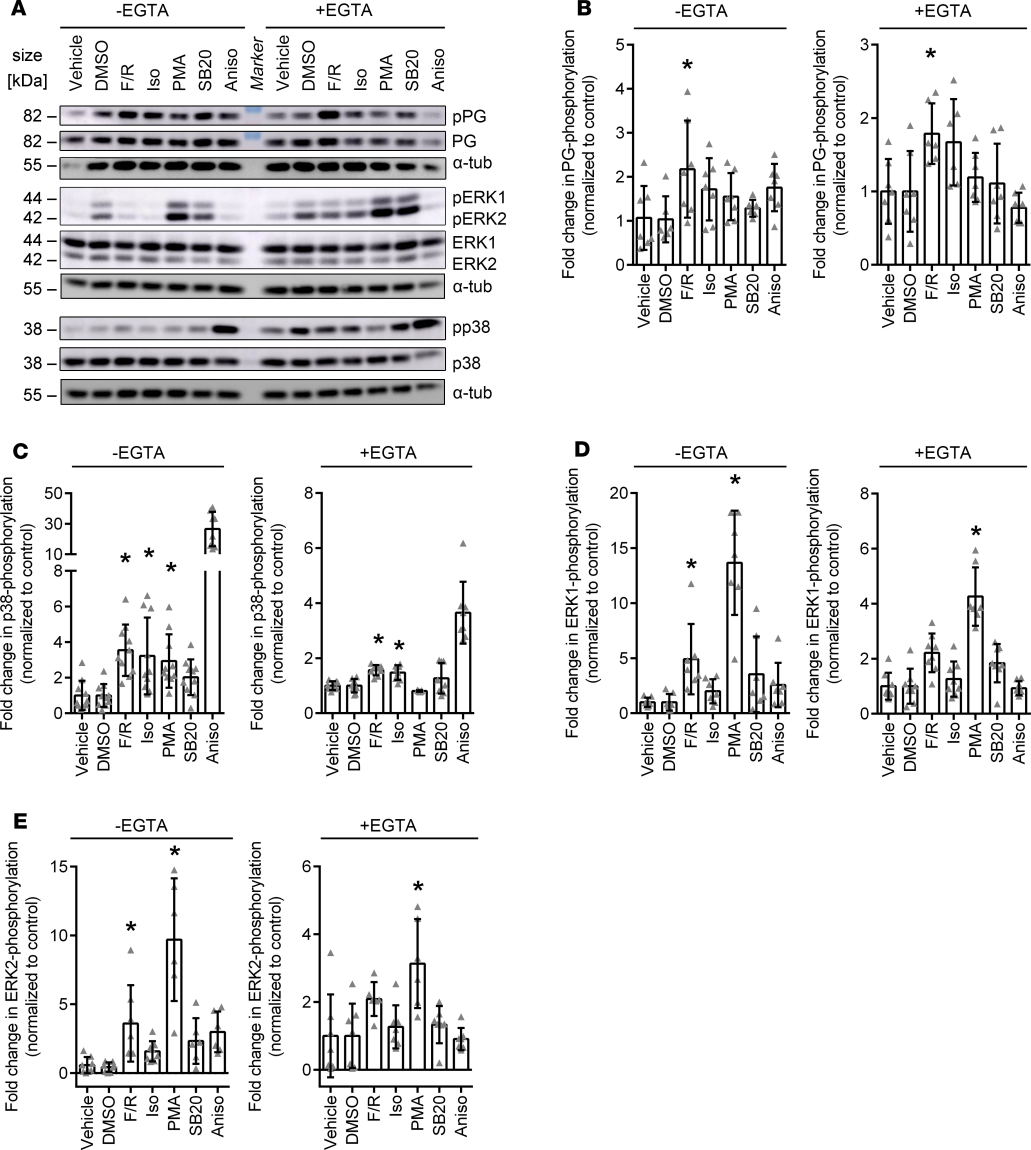
Phosphorylation of signaling proteins in basal and Ca^2+^-depleted conditions after treatments with mediators regulating cardiomyocyte cohesion. (**A**) Representative Western blot of HL-1 cells treated with F/R, Iso, PMA, SB20, or Aniso under basal and Ca^2+^-depleted conditions. DMSO serves as control for SB20. (**B–E**) Quantification of changes in phosphorylation levels of (**B**) PG, (**C**) p38MAPK, (**D**) ERK1, and (**E**) ERK2, as compared with the respective controls upon treatment with mediators. *P ≤ 0.05, 1-way ANOVA with Bonferroni correction, *n* = 6–9. In **C**, Aniso was excluded from the statistical analysis, as the effect was so strong that all other effects were not significant anymore with 1-way ANOVA.

To better understand whether the different signaling mechanisms regulating cardiomyocyte adhesion are to some extent interdependent, we performed Western blot analyses to assess the phosphorylation levels of PG and p38MAPK as well as ERK1/2 (Figure 2A). Under basal and Ca^2+^-depleted conditions, phosphorylation of PG at S665 increased significantly upon treatment with F/R (Figure 2B). As expected, p38MAPK was strongly activated upon treatment with Aniso. Moreover, p38MAPK activation upon treatment with F/R and Iso was detectable under basal as well as Ca^2+^-depleted conditions, whereas p38MAPK activation after PMA treatment was significant only under basal conditions (Figure 2C). Phosphorylation of ERK1/2 was enhanced significantly upon treatment with F/R as well as PMA under basal conditions. Following Ca^2+^ depletion, ERK2 phosphorylation was significantly upregulated upon PMA treatment only (Figure 2, D and E). No statistically significant increase of PG phosphorylation was observed upon treatment with Iso. The effects of p38MAPK inhibition on the phosphorylation status of the analyzed proteins were not statistically significant. Taken together, our data indicate that PG phosphorylation is specific for cAMP signaling, whereas ERK1/2 was activated when cAMP signaling and PKC activation enhanced cardiomyocyte adhesion.

### Inhibition of ERK1/2 signaling reverses the positive adhesiotropic effects of adrenergic signaling and PKC activation in HL1 cells.

**Figure 3:**
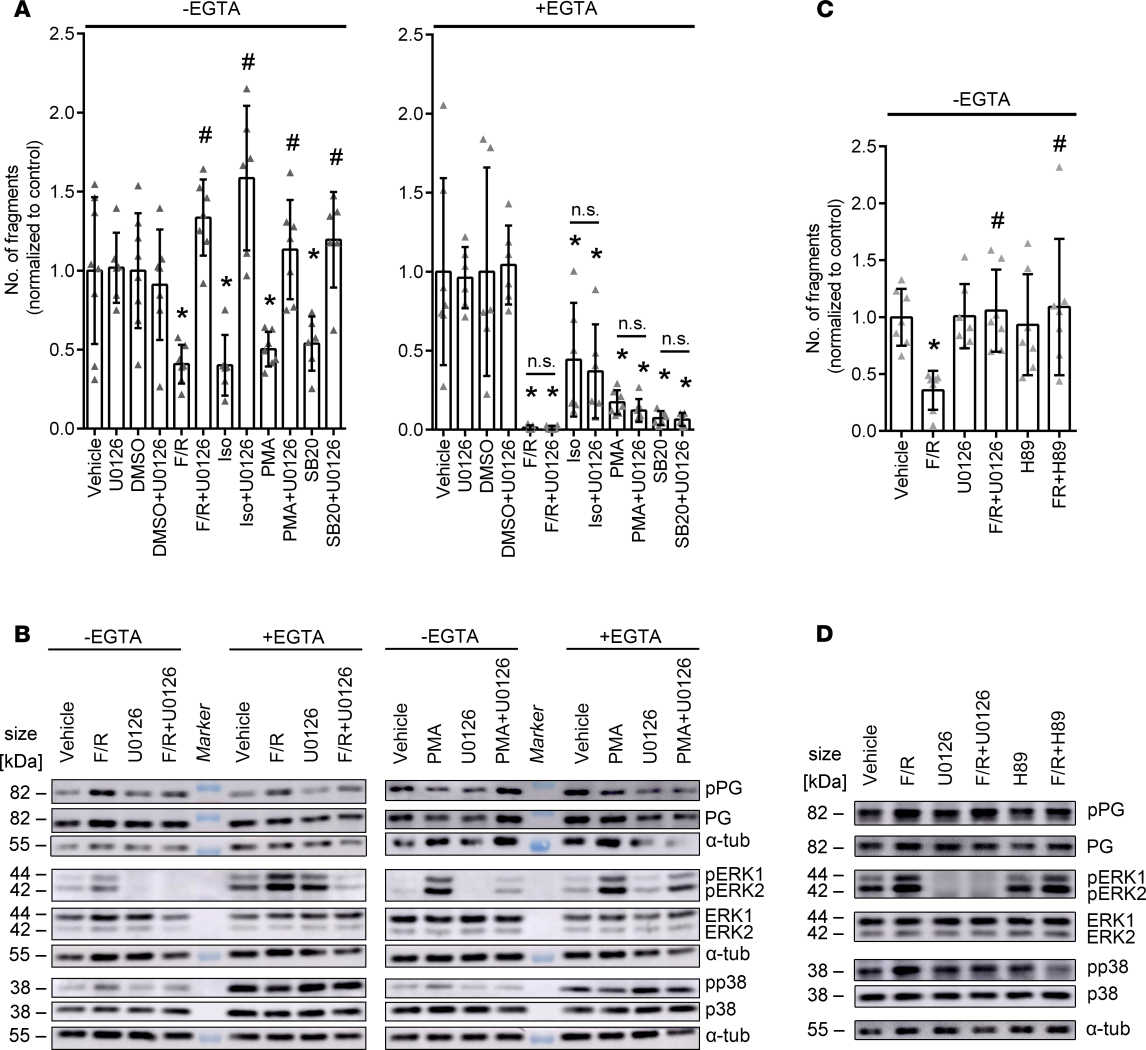
Inhibition of ERK1/2 alters cardiomyocyte cohesion in basal conditions and results in alteration of phosphorylation of PG, ERK1/2, and p38MAPK upon treatment with F/R and PMA. (**A**) Dissociation assays in HL-1 cells treated with F/R, Iso, SB20, or PMA, with and without U0126 under basal and Ca^2+^-depleted conditions. DMSO serves as control for SB20. *P ≤ 0.05 as compared with the respective control, ^#^*P* ≤ 0.05 as compared with the respective U0126-untreated condition, 1-way ANOVA with Holm-Šidák correction, *n* = 6. (**B**) Representative Western blot of HL-1 cells treated with F/R or PMA, with and without U0126 under basal and Ca^2+^-depleted conditions. *n* = 6. (**C**) Dissociation assays in HL-1 cells treated with F/R with and without U0126 or H89. *P ≤ 0.05 as compared with the vehicle control, ^#^*P* ≤ 0.05 as compared with F/R, 1-way ANOVA with Holm-Šidák correction, *n* = 6. (**D**) Representative Western blot of HL-1 cells treated with F/R with and without U0126 or H89. *n* = 6.

As we observed activation of ERK1/2 in both basal and hyperadhesive conditions by cAMP and PKC signaling, we performed dissociation assays using U0126, an inhibitor of MEK1/2, which is the upstream kinase of ERK1/2, to determine the role of ERK1/2 in cardiomyocyte adhesion, similar to studies in keratinocytes (32, 34). Interestingly, under basal conditions, but not under Ca^2+^-depleted conditions, inhibition of MEK1/2 by U0126 prevented the positive adhesiotropic effects of all mediators (Figure 3A). These data suggest that under basal conditions positive adhesiotropy is strictly dependent on MEK1/2 and thus on ERK1/2 phosphorylation, whereas hyperadhesion is ERK1/2 independent.

Next, Western blot analyses were performed under the same conditions and phosphorylation levels of PG, ERK1/2, and p38MAPK were assessed (Figure 3B and Supplemental Figure 3A). These experiments confirmed that U0126 efficiently blocked ERK1/2 phosphorylation under basal and Ca^2+^-depleted conditions. Interestingly, p38MAPK phosphorylation under these conditions was also blunted by U0126, indicating that activation of p38MAPK under conditions of enhanced adhesion is at least in part ERK1/2 dependent. Inhibition of MEK1/2 did not significantly affect F/R-induced PG phosphorylation, neither in basal nor in hyperadhesive conditions. Together, these data suggest that ERK1/2 activity is crucial for increased cardiomyocyte adhesion in HL-1 cells under basal, but not under Ca^2+^-depleted, conditions.

To get a better understanding of F/R-mediated positive adhesiotropy, i.e., to assess whether ERK1/2 and PG phosphorylation are 2 separate pathways activated by enhanced cAMP, we performed dissociation assays using H89, a PKA inhibitor (Figure 3C). Our data show that PKA inhibition, similar to MEK1/2 inhibition, prevented F/R-mediated positive adhesiotropy. We then performed Western blot analyses of the same conditions and found that PKA inhibition does not inhibit F/R-mediated ERK1/2 phosphorylation but does prevent PKA-mediated phosphorylation of PG at S665 (Figure 3D and Supplemental Figure 3B). Taken together, our data suggests that both, PG phosphorylation at S665 as well as ERK1/2 activation are needed for the F/R-mediated positive adhesiotropy in cardiomyocytes.

### Inhibition of MEK1/2 decreases junctional localization of DSG2.

**Figure 4:**
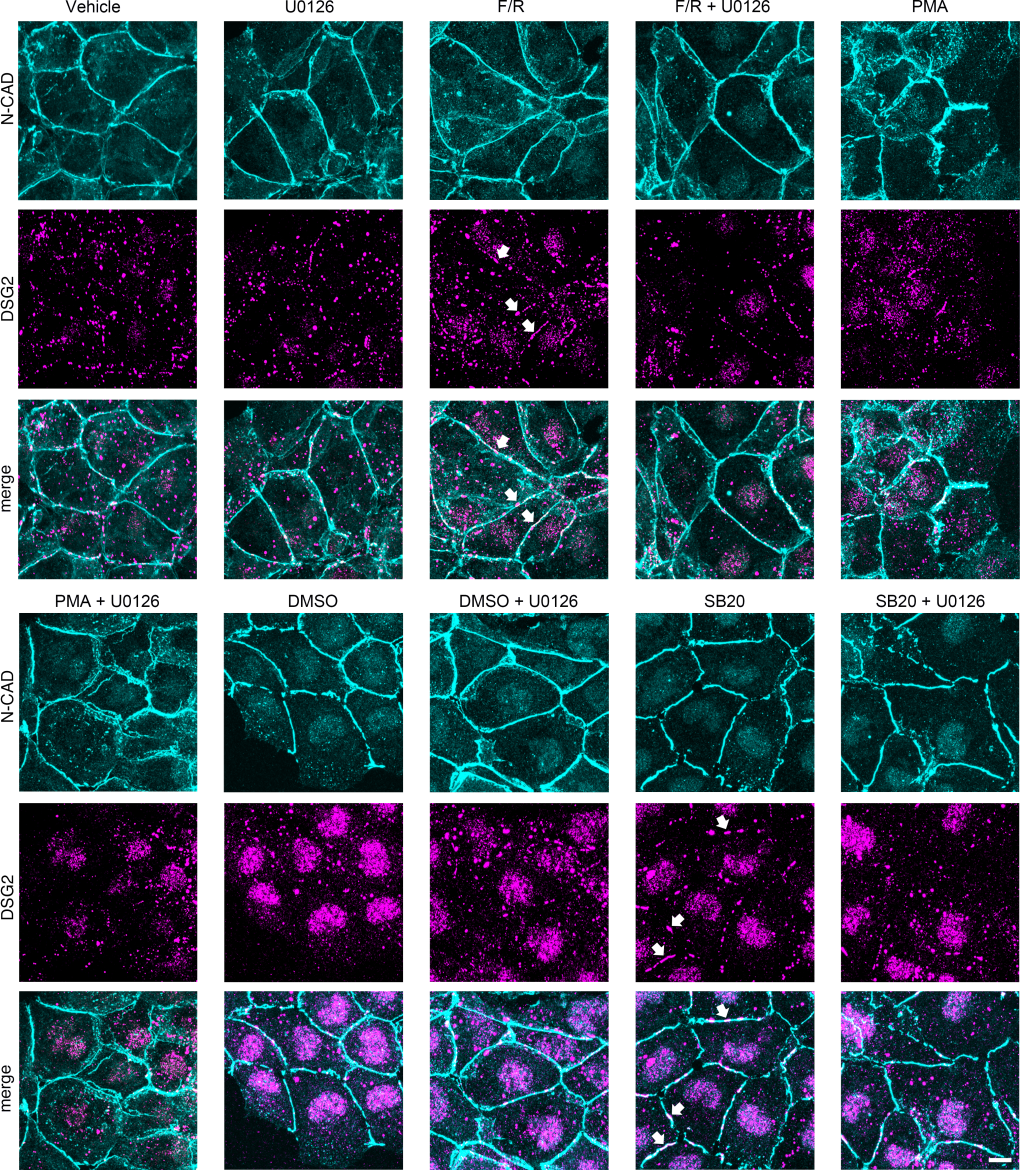
ERK1/2 inhibition caused alteration of DSG2 localization. Immunostainings of HL-1 cells treated with F/R, PMA, or SB20, with and without U0126, stained for N-CAD and DSG2 under basal conditions. DMSO serves as control for SB20 treatment. Arrows indicate increased localization of DSG2 in the membrane. Scale bar: 10 μm. *n* = 6.

To determine whether the observed enhanced cardiomyocyte adhesion was due to altered localization of DSG2 or N-CAD in the membrane, we performed immunostaining for DSG2 and N-CAD in HL-1 cells and quantified the effects. Under all conditions, N-CAD was distributed continuously along cell junctions, whereas DSG2 localization was confined to punctuate stainings along the cell membrane (Figure 4). Treatment with U0126 or PMA alone did not affect, whereas F/R significantly enhanced, DSG2 colocalization with N-CAD at the membrane, the latter of which is in agreement with previous studies (22, 35). Inhibition of p38MAPK by SB20, similar to F/R treatment, led to increased localization of DSG2 to the cell borders. Treatment with a positive adhesiotropic mediator in combination with U0126 resulted in reduced DSG2 localization at the membrane, as compared with samples treated with the mediator alone (Figure 4 and Supplemental Figure 4). Our results suggest that the positive adhesiotropy by adrenergic signaling and p38MAPK inhibition occurs via ERK1/2-dependent DSG2 membrane localization.

### In cultured cardiomyocytes positive adhesiotropy is dependent on the presence of PG, DP, and DSG2.

**Figure 5:**
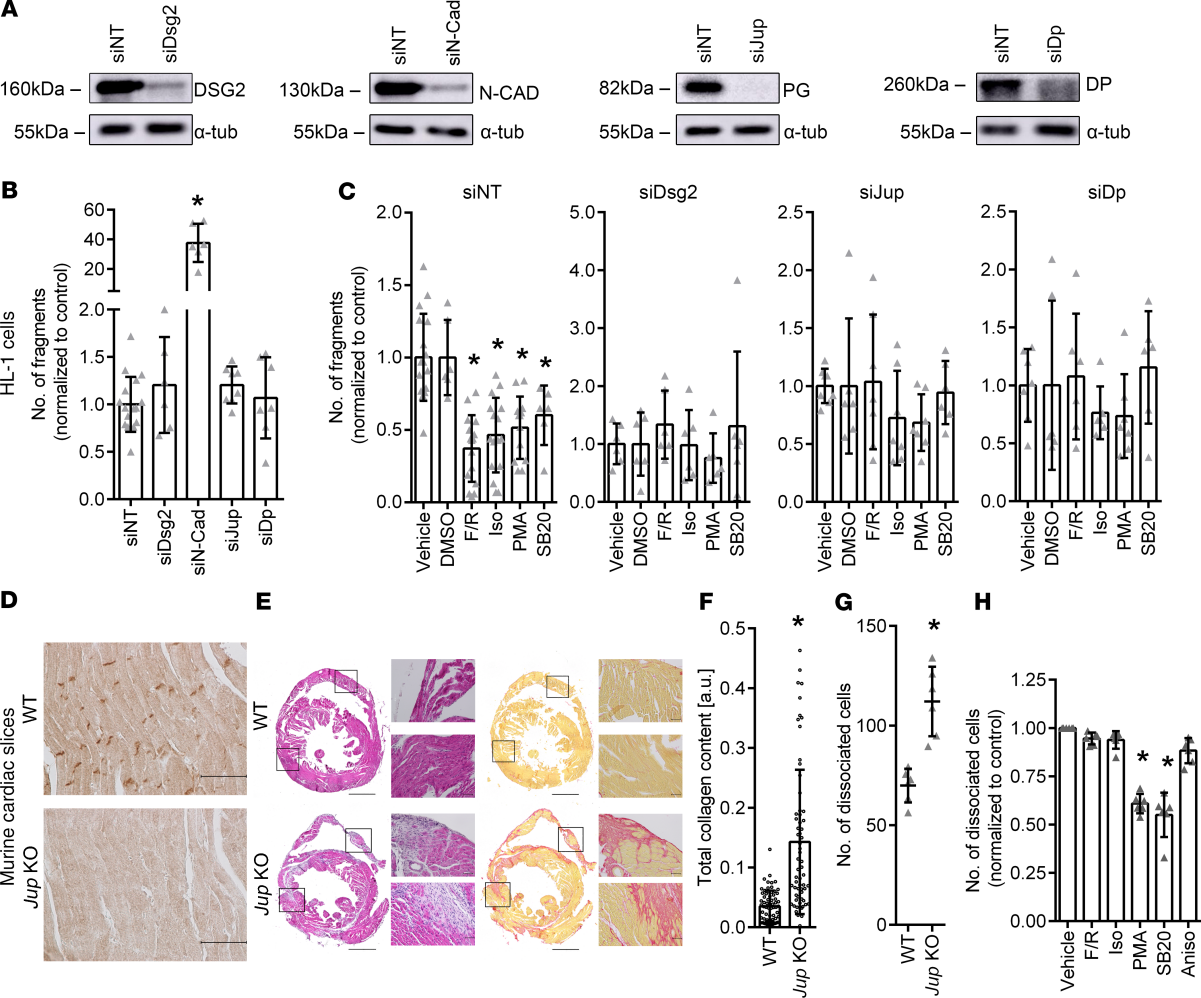
Effect of *Dsg2*, *N-Cad*, *Jup*, or *Dp* knockdown on F/R-, Iso-, PMA-, or SB20-mediated increase in cardiomyocyte cohesion. (**A**) Representative Western blots confirming decreased levels of DSG2, N-CAD, PG, and DP after siRNA-mediated knockdown. (**B**) Dissociation assay under basal conditions following siNT, siDsg2, siN-Cad, siJup, and siDp treatments. *P ≤ 0.05, 1-way ANOVA with Holm-Šidák correction, *n* = 6. (**C**) Dissociation assays showing fold changes in fragments as compared with the respective controls in HL-1 cells following siNT, siDsg2, siJup, or siDp treatments and administration of F/R, Iso, PMA, or SB20. DMSO serves as control for SB20. *P ≤ 0.05, 1-way ANOVAs with Holm-Šidák correction, *n* = 6. (**D**) Immunohistochemistry of ventricular cardiac slices obtained from WT and PG-deficient mice (*Jup*-KO) stained for PG protein. Scale bar: 50 μm. *n* = 6. (**E**) H&E and Picrosirius red stainings of cardiac slices obtained from WT and *Jup*-KO mice. Scale bar: 1 mm; 50 µm (high-magnification views). (**F**) Total collagen content of cardiac slices obtained from WT and *Jup*-KO mice. *P ≤ 0.05, Student’s *t* test with Welch correction, *n* = 6. (**G**) Dissociation assays in murine cardiac slice cultures comparing the number of dissociated cells in WT and *Jup*-KO cardiac slices. *P ≤ 0.05, unpaired Student’s *t* test, *n* = 6. (**H**) Dissociation assays in murine cardiac slices obtained from *Jup*-KO mice following F/R, Iso, PMA, SB20, and Aniso treatments. *P ≤ 0.05, 1-way ANOVA with Holm-Šidák correction, *n* = 6.

Having shown that adrenergic signaling, PKC activation, and p38MAPK inhibition can induce positive adhesiotropy, we further explored the possible desmosomal protein targets of these pathways. Therefore, we knocked down *Jup* (the gene coding for PG), *Dp*, *Dsg2*, and *N-Cad* by siRNA and performed dissociation assays; nontarget siRNA (siNT) transfections served as controls. Western blot analyses were used to determine the knockdown efficiency (Figure 5A). We observed no change in adhesion in HL-1 cells upon knockdown of *Jup*, *Dp*, or *Dsg2*. However, cells lacking N-CAD had strongly decreased stability of the cellular monolayer (Figure 5B), which fell apart even without mechanical stress after dissociation from the well bottom, indicating that loss of PG, DP, and DSG2, but not of N-CAD, can be compensated. Next, cells with *Jup*, *Dp*, or *Dsg2* knockdown were treated with positive adhesiotropic mediators. The positive adhesiotropic effect of F/R, Iso, PMA, and SB20 was dependent on the presence of PG, DP, and DSG2 (Figure 5C). In our experimental setting, we could not quantify the effects of the mediators in cells where *N-Cad* was knocked down, as the fragmentation of the cellular monolayer was too strong, and the variability was so high, that no reliable data could be obtained.

Our data indicate that N-CAD is crucial for cardiomyocyte adhesion, while knockdown of *Jup*, *Dp*, and *Dsg2* can be compensated in HL-1 cells. Furthermore, the effect of adrenergic signaling, PKC activation, and p38MAPK inhibition is strictly dependent on the presence of PG, DP, and DSG2 in HL-1 cells.

### In murine ventricular cardiac slice cultures from PG-deficient mice, activation of PKC and p38MAPK inhibition, but not adrenergic signaling, enhances cardiomyocyte adhesion.

To transfer our findings from the cell culture to a disease model, we performed dissociation assays with ventricular murine cardiac slice cultures obtained from PG-deficient mice, which have an AC phenotype with fibrous replacement of cardiac tissue. Immunohistochemical analysis of cardiac tissue from WT and KO mice showed that PG was located in the ICD in WT mice. At the same time, there was no PG staining in the KO mouse (Figure 5D). Picrosirius red staining, as well as H&E staining, confirmed increased fibrosis in hearts from PG-deficient mice with increased collagen content (Figure 5, E and F). In dissociation assays performed with cardiac slices obtained from 6- to-8-week-old WT and PG-deficient mice, we observed a significant decrease in cellular adhesion in PG-deficient mice (Figure 5G). As we used collagenase and dispase for the dissociation assays and the PG-deficient mice exhibited fibrosis (Figure 5, E and F), the enzymes could dissociate fibroblasts and other cell types apart from cardiomyocytes. In order to avoid this discrepancy, we counted only viable cylindrical shaped cardiomyocytes, thereby minimizing the chances of counting dissociated fibroblasts and other cell types. In contrast to our findings from cell culture experiments, we observed that PKC activation and p38MAPK inhibition, but not adrenergic signaling, induced positive adhesiotropy in PG-deficient mice (Figure 5H).

Our data indicate that, in murine cardiac slices, positive adhesiotropy induced by PKC and p38MAPK signaling is independent of PG and is still inducible despite presence of fibrous alterations similar to patients with advanced disease. In contrast, positive adhesiotropy mediated by adrenergic signaling is strictly dependent on PG.

## Discussion

In this study, we have elucidated that a hyperadhesive state of cardiomyocytes, in addition to basal cardiomyocyte adhesion, can be induced by adrenergic signaling, PKC activation, and p38MAPK inhibition and that basal cardiomyocyte adhesion, but not hyperadhesion, is ERK1/2 dependent. In addition, we show that an activation of p38MAPK negatively regulated cardiomyocyte adhesion in HL-1 cardiomyocytes and may be involved in the mechanisms by which Ca^2+^ depletion induced loss of cell adhesion. On the other hand, we found that positive adhesiotropy in response to adrenergic signaling seems to be dependent on PG and paralleled by PG phosphorylation. In contrast, PKC and p38MAPK regulate cardiomyocyte adhesion independent of PG in murine cardiac slice cultures. In cultured cardiomyocytes, DP, PG, and DSG2 seem to be crucial for positive adhesiotropy induced by adrenergic signaling, p38MAPK inhibition, or PKC activation. As adrenergic signaling is known to exist under physiologic conditions, we conclude that adrenergic signaling stabilizes cardiomyocyte adhesion under physiologic conditions, whereas this protective mechanism might be offset in PG-dependent AC, which may contribute to disease pathogenesis.

Ca^2+^ signaling is tightly regulated in cardiomyocytes, where intracellular and extracellular Ca^2+^ levels constantly change, and it is thus possible that under the physiological state of cardiomyocytes, a phenomenon of hyperadhesion exists. In fact, it was proposed that most desmosomes of intact epidermis are hyperadhesive (19–21). In this study, we showed that the chelation of extracellular Ca^2+^ leads to a decreased intercellular adhesion of cultured cardiomyocytes. It must be noted that Ca^2+^ depletion by chelation is unphysiological. Nevertheless, this approach is important to characterize the signaling mechanisms regulating cardiomyocyte adhesion. In addition, these signaling mechanisms may be involved in pathogenesis or therapy of AC and other cardiac disorders, especially as the decreased adhesion upon Ca^2+^ depletion could be overcome by adrenergic signaling, p38MAPK inhibition, or PKC activation in murine cardiac slice cultures and HL-1 cells. Furthermore, deregulation of Ca^2+^ signaling is known to be causative for several arrhythmic disorders (36–38), indicating that our findings might be of interest when studying other cardiomyopathies.

In line with findings from the pemphigus field, we showed here that p38MAPK inhibition is beneficial for cardiomyocyte adhesion, while p38MAPK activation decreases it (39–41). However, in contrast to keratinocytes (42–44), PKC activation seems to be beneficial for cellular adhesion, both in cultured cardiomyocytes as well as in murine cardiac slices. The different effects of the PKC signaling pathway can be due to differences in Ca^2+^ homeostasis between keratinocytes (45) and cardiomyocytes (46, 47).

The role of p38MAPK in the regulation of cardiomyocytes seems to be complex: it is activated upon adrenergic signaling and PKC activation, both of which induce positive adhesiotropy. On the other hand, direct p38MAPK activation by Aniso reduced cellular adhesion, whereas p38MAPK inhibition led to positive adhesiotropy. This suggests that p38MAPK activation is not required for adrenergic and PKC signaling to enhance cardiomyocyte adhesion and that the destabilizing effects of p38MAPK activation are outbalanced by the stabilizing effects of these pathways (48). Interestingly, it was shown that, in end-stage failing human myocardium, reduced activation of p38MAPKα, which is the predominant cardiac isoform, occurs before end-stage heart failure (49). Therefore, we believe that further studies are warranted to delineate the complete molecular mechanism of p38MAPK-mediated regulation of cardiomyocyte adhesion.

Our major finding in this study is that positive adhesiotropy in cardiomyocytes under basal conditions is strongly dependent on ERK1/2 activation, as ERK1/2 inhibition reduced the positive adhesiotropic effects of adrenergic signaling, PKC activation, and p38MAPK inhibition. Immunostaining of DSG2 in HL-1 cells showed that p38MAPK inhibition, as well as the cAMP-mediated increase in cardiomyocyte adhesion, enhanced localization of DSG2 at the membrane, a process which depends on activation of ERK1/2. In a previous study, Bueno et al. used transgenic mice overexpressing MEK1 and showed that the MEK1/ERK1/2 signaling pathway stimulates a physiologic hypertrophy, which augmented cardiac function without lethality (50). In addition, the same group also demonstrated that MEK1/ERK1/2 signaling protects the heart from apoptotic insults in vivo. Similar to these observations, our findings might be of great importance for therapeutically targeting AC. Recent data indicate that ERK1/2 is activated in AC (51). Mutations in DP cause ERK1/2 activation, which facilitated tachycardia via depletion of β1D integrin. Therefore, it is possible that a rescue mechanism to stabilize cardiomyocyte adhesion via ERK1/2 contributes to arrhythmia. In our previous (22) and the current study, we found that adrenergic signaling leads to PG phosphorylation at S665 and thereby positive adhesiotropy. However, to our surprise, in this study, upon inhibition of ERK1/2, adrenergic signaling–mediated positive adhesiotropy was completely abolished, although PG phosphorylation was undisturbed in HL-1 cells. Furthermore, in *Jup*-KO mice adrenergic signaling–mediated positive adhesiotropy was completely abolished, as was observed before (22). Similar effects were observed in HL-1 cells, where knockdown of *Jup* annihilated adrenergic signaling–mediated positive adhesiotropy. Our data show that both PG phosphorylation and ERK1/2 activation are required for adrenergic signaling–mediated positive adhesiotropy under basal conditions. In contrast, hyperadhesion is ERK1/2 independent, as the administration of U0126 did not alter hyperadhesion in HL-1 cardiomyocytes, indicating that the mechanisms of positive adhesiotropy and hyperadhesion differ at least in part.

In order to find desmosomal protein targets for the observed differences in cardiomyocyte adhesion by the above mediators, we performed knockdown experiments using HL-1 cells. In dissociation assays performed in HL-1 cells, knockdown of *Dsg2* or *Jup* or *Dp* led to the loss of observed beneficial effects of adrenergic signaling, PKC activation, and p38MAPK inhibition. These data prove that the presence of all desmosomal components is essential for positive adhesiotropy. We next evaluated our findings using the AC disease mouse model with a cardiac-specific KO of *Jup*. These mice develop an AC pathology, where fibrosis was evident by 6–8 weeks, which allows us to mimic the situations in patients with the progredient disease. Moreover, this model is informative on whether secondary alterations completely abolish the regulation of adhesion. Despite fibrosis, p38MAPK inhibition and PKC activation were still effective in increasing cardiac adhesion in ventricular cardiac slice cultures from PG-deficient mice. Thus, altering these pathways might be a possible therapeutical approach to stabilize cardiomyocyte adhesion in the future, even after disease manifestation.

To date, the treatment of AC is based on symptomatic relief with treatment options being beta blockers, antiarrhythmic agents (class 1 and 3), heart catheter ablation, or sympathetic denervation (52). At first glance, our study might put into question or even contraindicate treatment with beta blockers, as in our experimental settings treating cultured cardiomyocytes or WT murine cardiac slice cultures with the β-adrenergic stimulator Iso led to an increase in cardiomyocyte adhesion. This is not the case, especially since beta blockers, besides ventricular cardiomyocytes, also target the conduction system to reduce arrhythmia. In addition, the beneficial effects of beta blockers for patients with AC are known, and there are studies suggesting adverse effects of adrenergic signaling for patients suffering from cardiomyopathies (53). Therefore, we performed dissociation assays in HL-1 cells using beta blockers. We did not observe any changes in cardiomyocyte cohesion upon beta blocker treatment. These data show that beta blockers alone do not have an effect on cardiomyocyte cohesion in HL-1 cells. Future studies are necessary to investigate the effects of adrenergic stimulation and beta blockers on adhesiotropy, especially with an AC model, both under basal and Ca^2+^-depleted conditions. In addition, chronic or high-dose administration of Iso is known to induce hypertrophy and myocardial infarction–like symptoms in mouse and rat hearts (54, 55). Furthermore, and more importantly, in our PG-deficient AC disease model, as well as in HL-1 cells, where genes coding for desmosomal proteins were knocked down, we could not observe a positive adhesiotropic effect of Iso. This puts into question whether adrenergic signaling would be an effective way to treat potential patients and indicates that it is only beneficial in healthy cardiomyocytes.

The limitation of our current study is the usage of PG-deficient mice as AC disease model, which is a disease that can be caused by several genetic defects, including, but not limited to PG mutations. Apart from genes coding for the desmosomal proteins, such as PG, DP, PKP2, DSG2, and DSC2, genes coding for proteins of the nuclear envelope, intermediate filaments, growth factors, ion channels, or area composita can be causative (4). The effect of adrenergic signaling, PKC activation, and p38MAPK inhibition on AC models with other causative mutations should be part of future investigations. Another limitation of using this mouse model to study cardiomyocyte cohesion was the presence of fibrosis. For dissociation assays, we used dispase and collagenase, which can break the extracellular matrix and thereby dissociate fibroblasts and other cell types in addition to the cardiomyocytes. Therefore, we counted only cylindrical shaped cardiomyocytes to avoid confounding the results and maximized the chances to analyze cardiomyocyte cohesion. Using HL-1 cardiomyocytes for cardiac studies has the advantage that HL-1 cells are immortalized cells, which can be grown to confluent cell monolayers. For studies in cardiomyocyte cohesion, where dissociation assays are required, this is absolutely critical. However, the cells represent more of a mixture of embryonic and adult atrial cardiomyocytes. Nevertheless, they have cardiac-specific junctions expressed in the ICD, undergo spontaneous contraction, and express cardiac-specific genes, including α-MHC, α-cardiac actin, desmin, connexin 40 and 43, and several voltage-dependent channels, which are expressed in an adult cardiomyocyte. As we reproduced most of the findings in HL-1 cells with murine cardiac slices, we believe that performing experiments in HL-1 cells in combination with cardiac slice cultures could overcome the shortcomings of both models.

**Figure 6:**
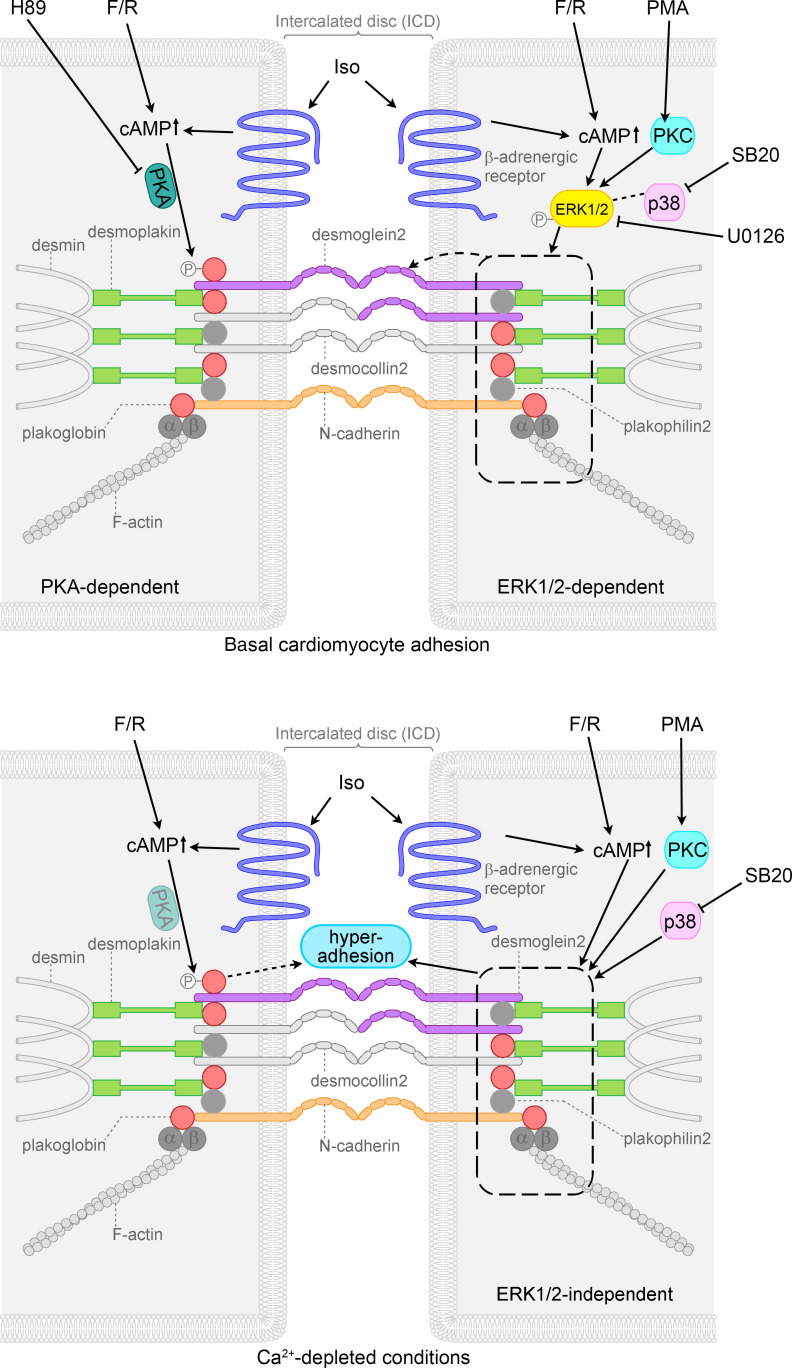
Schematic overview of signaling mechanisms involved in cohesion in HL-1 cardiomyocytes. Adrenergic signaling (F/R and Iso), PKC activation (PMA), and p38MAPK inhibition (SB20) enhanced basal cardiomyocyte cohesion, referred to as positive adhesiotropy, in an ERK1/2-dependent manner. Positive adhesiotropy is possibly achieved through alterations in the interactions of the desmosomal proteins PG, PKP2, and DP (represented by dashed rectangle) that eventually lead to increased DSG2 translocation to the cell borders (represented by dashed arrow) and finally to positive adhesiotropy. On the other hand adrenergic signaling also acts via PKA-dependent PG phosphorylation at S665 and thereby increases basal cardiomyocyte cohesion. Under Ca^2+^-depleted conditions, adrenergic signaling, PKC activation, and p38MAPK inhibition lead to hyperadhesion independent of ERK1/2 (represented by dashed rectangle and straight arrow). Under the same conditions adrenergic signaling also induces PG phosphorylation, probably via PKA (shaded PKA), and thereby hyperadhesion (dashed arrow).

In summary, as illustrated in Figure 6, our data suggest that positive adhesiotropy and hyperadhesion are induced by adrenergic signaling, PKC activation, and p38MAPK inhibition, the former of which depend on ERK1/2 activation. Adrenergic signaling, on the other hand, requires both PKA-dependent PG phosphorylation at S665 and ERK1/2 activation for positive adhesiotropy under basal conditions. In contrast, in hyperadhesion the role of PKA is not clear yet, but PG phosphorylation by adrenergic signaling seems to be important also. p38MAPK inhibition and PKC activation can restore the reduced cardiomyocyte adhesion in our PG-deficient AC mouse model. We believe that these observations will be of therapeutic benefit in treating AC in the future.

## Methods

For full information on materials and methods, please refer to the Supplemental Methods.

### Cell culture.

Immortalized murine atrial myocyte (HL-1 cells) provided by William Claycomb (LSU Health Sciences Center, New Orleans, Louisiana, USA) were cultured in supplemented Claycomb medium at 37°C and 5% CO_2_ in gelatin- and fibronectin-coated T75 flasks. For experiments, cells were cultured in coated well plates in fully supplemented medium without norepinephrine.

### Mediators and reagents.

Concentrations and functions of the mediators used in this study are provided in Table 1. When performing experiments with β-adrenergic receptor blockers, cells were cultured in medium containing norepinephrine.

### Murine heart slice culture.

Mice were purchased from The Jackson Laboratory. Six- to eight-week-old, age and sex-matched littermates of *Jup* (junctional PG, gene coding for PG) WT and KO mice (22) were used for experiments. Murine slice cultures were obtained as described before (22). In brief, mice were sacrificed by cervical dislocation, hearts were immediately placed in precooled oxygenated cardiac slicing buffer, embedded in low-melt agarose, and cut with a LeicaVT1200S vibratome (Leica Biosystems). For dissociation assays, slices were washed gently with HBSS, transferred to prewarmed cardiac slices medium, and incubated for 1 hour with indicated mediators at 37°C, 5% CO_2_.

### Immunostaining.

For immunostainings, cells were seeded on glass coverslips. After treatment, cells were fixed with paraformaldehyde (PFA), washed, permeabilized with 0.1% Triton X-100, and blocked with bovine serum albumin and normal goat serum in a wet chamber. Following primary antibody and species-matched, fluorophore-coupled secondary antibody incubations, slides were mounted using NPG and analyzed using a Leica SP5 confocal microscope with a ×63 oil objective and LAS-AF software. Z-scans were performed at 0.25 μm thickness, spanning the whole cell volume. Colocalization of proteins was analyzed with ImageJ software (NIH): regions of interest were selected for N-CAD staining and were transferred to the corresponding DSG2 image. The percentage of the area where pixel values were not equal to 0, after removing the background, was measured as shown in Supplemental Figure 1.

### Histological analysis.

Hearts were dissected and fixed in 4% PFA, dehydrated, and embedded in paraffin.

Global heart morphology was determined from transversal 10 μm deparaffinized sections stained with H&E. Fibrosis was detected with Picrosirius red stain. Samples were analyzed in a Keyence Biozero BZ-800 microscope. Hearts from 6 different animals were quantified for each experimental group.

For immunohistochemical analyses, sections (10 μm) were cut, mounted, deparaffinized in xylene, and rehydrated through an ethanol series. After treatment at 95°C in a citric acid–based antigen unmasking solution, sections were incubated in 0.3% H_2_O_2_ in methanol. Sections were permeabilized using 0.2% Nonidet, rinsed, blocked, and incubated overnight with mouse anti-PG. After washing, sections were incubated with the diluted biotinylated secondary antibody, rinsed, and incubated with VECTASTAIN Elite ABC reagent. After final washing, sections were developed in a peroxidase substrate solution, mounted, and analyzed in a Keyence Biozero BZ-800 microscope. Hearts from 6 different animals were quantified for each experimental group.

### Dissociation assays in HL-1 cells.

Under basal conditions, after respective treatments, cells were washed with HBSS, treated with Liberase-DH and Dispase II, and incubated at 37°C until the cell monolayer detached from the wells. Then, the enzyme mix was carefully removed from the wells and replaced by HBSS. Mechanical stress was applied by pipetting up and down 4 times with an electrical pipette. When dissociation assays were performed after siRNA treatment, mechanical stress was applied by shaking on an orbital shaker for 5 minutes at 1.31*g* (1250 rpm, Stuart microtitre plate shaker SSM5). Images of the wells were taken to count the number of fragments. The amount of fragmentation was taken as an indirect measurement for intercellular adhesion, with an increased fragmentation of the monolayer being a sign for decreased intercellular adhesion.

Under Ca^2+^-depleted conditions, cells were detached from the wells with Liberase-DH and Dispase II and supplemented with Claycomb medium along with the mediators for 60 minutes. After incubation, the medium was replaced by fully supplemented, ethylene glycol-bis(β-aminoethyl ether)-N,N,N′,N′-tetraacetic acid–containing (EGTA-containing) Claycomb medium along with the mediators for 90 minutes. Finally, the cell monolayers were mechanically agitated, as explained above, and the resulting fragments were imaged and counted using ImageJ software (NIH).

### Dissociation assays in murine cardiac slices.

When performing dissociation assays with murine cardiac slice cultures, slices were treated and washed with HBSS before Dispase II was added together with Liberase-DH. Slices were incubated at 37°C, 5% CO_2_ for 25 minutes. Mechanical stress was applied using an electrical pipette, and cells were stained with MTT. After a settling time of 5 minutes, medium containing cell debris and detached cells was filtered using a 70 μm nylon membrane, and the number of viable dissociated cardiomyocytes was counted with an inverted microscope (Axio, Carl Zeiss) and taken as an indirect measurement for intercellular adhesion. To control variations due to different slice size or location in the ventricle, consecutive slices for control and treatment were used, respectively, and the result of a slice was compared with the respective control slice.

### Western blot analyses.

After respective treatments, cells were washed with PBS, kept on ice, scraped into SDS-lysis buffer, and sonicated. Protein concentration was determined using the Pierce Protein Assay Kit. Prior to electrophoresis, samples were denatured in Laemmli buffer. Proteins were loaded on SDS-PAGE gels together with a PageRuler Plus Prestained Protein Ladder. After gel electrophoresis, proteins were transferred to a nitrocellulose membrane using the wet-blot method. Following blocking and incubation with primary antibodies and horseradish peroxidase–conjugated secondary antibodies, membranes were washed and developed using the ECL method with an Amersham Imager 600 (General Electric). Quantification was performed using ImageJ software (NIH). See complete unedited blots in the supplemental material.

### siRNA knockdown.

Cells were transfected with siRNA 24 hours after seeding. Therefore, the transfection reagent, containing OptiMEM, RNAiMAX, and the ON-Target siRNA (Dharmacon) was added according to the manufacturer’s protocol. Twenty-four and seventy-two hours after transfection, medium was changed. Experiments were performed 84 hours after transfection. Knockdown efficiency was confirmed by Western blot.

### Imaging.

Images were processed with Adobe Photoshop CS5 and ImageJ software (NIH).

### Statistics.

Statistical analyses were performed using GraphPad Prism 8. Two-tailed Student’s *t* tests and 1-way ANOVA analyses with post hoc tests were applied accordingly after outlier removal and are explained in figure legends. Outliers were removed with GraphPad Prism 8 outlier removal, with a likelihood of 1%. Data are represented as mean ± SD. For dissociation assays in HL-1 cells, the number of fragments was normalized to the respective controls. For dissociation assays as well as for quantification data for Western blots in HL-1 cells, single control values were normalized to the average control value of all experiments. In mice, the number of dissociated cells was normalized to the respective control slice of the same mouse, as mouse-to-mouse variability was too high to normalize the treated mice group with the control group. Significance was assumed at *P* ≤ 0.05.

### Study approval.

Animal handling, breeding, and sacrifice were performed with approval of protocols by the regional government of Upper Bavaria (Gz., 55.2-1-54-2532.139.2014), which complies with the guidelines from Directive 2010/63/EU of the European Parliament.

## Author contributions

MS, ST, EK, and TW acquired and analyzed the data. BG, CS, and SY analyzed the data. MS, SY, and JW drafted the manuscript. CS, SY, and JW made critical revision of the manuscript for important intellectual content. SY and JW handled supervision and designed the research.

## Supplementary Material

Supplemental data

## Figures and Tables

**Table 1:**
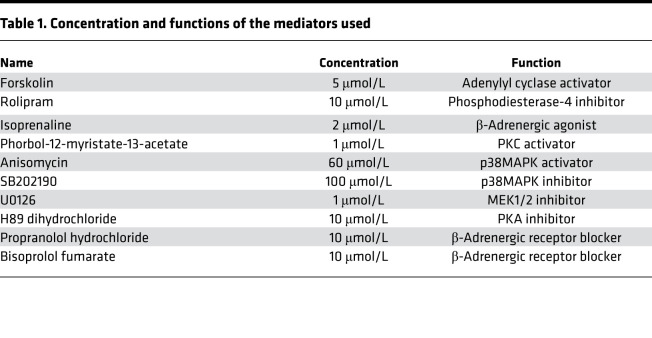
Concentration and functions of the mediators used

## Version 1 08/25/2020

In-Press Preview

## Version 2 09/17/2020

Electronic publication
