# Supplementary material for: Cardiomyocyte adhesion and hyperadhesion differentially require ERK1/2 and plakoglobin
Source: JCI Insight. 2020 Sep 17;5(18):e140066. doi: 10.1172/jci.insight.140066 (PMC7526536; doi:10.1172/jci.insight.140066)
Supplement: Supplemental data [file jciinsight-5-140066-s227.pdf]

Supplementary Material to Research Article

Cardiomyocyte adhesion and hyper-adhesion differentially require ERK1/2 and plakoglobin.

Maria Shoykhet[1], Sebastian Trenz[1], Ellen Kempf[1], Tatjana Williams[2], Brenda Gerull[2], Camilla Schinner[1+], Sunil Yeruva[§1] and Jens Waschke[§1]

[1]Faculty of Medicine, Ludwig-Maximilians-University (LMU) Munich, Munich, Germany

[2]Comprehensive Heart Failure Center and Department of Internal Medicine I, University Hospital Würzburg, Würzburg, Germany

[+] Current address: Department of Biomedicine, University of Basel, Basel, Switzerland

§ **Corresponding authors:**

Sunil Yeruva and Jens Waschke, Institute of Anatomy and Cell Biology, Department I, Ludwig-Maximilians-University, Pettenkoferstraße 11, 80336 Munich, Germany; Phone: +49-89-2180-72610; Fax: +49-89-2180-72602; sunil.yeruva@med.uni-muenchen.de; jens.waschke@med.uni-muenchen.de

1. Supplementary Material and Methods

1.1.Cell culture

Immortalized murine atrial myocyte cells (HL-1 cells) were cultured in Claycomb medium (#51800C) with 10% foetal bovine serum (FBS, #F2442), 100 µmol/mL norepinephrine (NE, #1468501), 100 µg/mL penicillin/streptomycin (AppliChem #A1837 and #A1852) and 2 µmol/mL L-glutamine (#G7513) at 37°C and 5% CO_2 in T75 flasks coated with 0.02% gelatine (#G1890) and 25 µg/mL fibronectin (#F1141). If not stated otherwise, all reagents were purchased from Sigma-Aldrich. Depending on cell passage and experimental design, cells were seeded at 75000-150000 cells/cm² for experiments in 25 µg/ml fibronectin and 0.02% gelatine-coated cell culture plates and supplemented Claycomb medium without NE. When performing experiments with β-adrenergic receptor blockers, cells were cultured in fully supplemented Claycomb medium with NE.

1.2.Mediators and reagents

All activating mediators were incubated for 60 minutes, while all inhibitors were added 30 minutes prior to adding mediators, unless otherwise stated. Concentrations of mediators and inhibitors used in this study were provided in Supplemental Table 1.

Supplemental Table 1: Concentration of mediators and inhibitors used for this study.

**Name**	**Company**	**Catalog.No**	**Concentration**
Forskolin (F)	Sigma-Aldrich	#F3917	5 µmol/L
Rolipram (R)	Sigma-Aldrich	#R6520	10 µmol/L
Isoprenaline (Iso)	Sigma-Aldrich	#I5627	2 µmol/L
Phorbol-12-myristat-13-acetate (PMA)	Sigma-Aldrich	#P1585	1 µmol/L
Anisomycin (Aniso)	Sigma-Aldrich	#10522	60 µmol/L
SB202190 (SB20)	Cell Signaling	#8158S	100 µmol/L
U0126	Sigma-Aldrich	#L9908	1 µmol/L
H89 dihydrochloride (H89)	Santa Cruz	#sc-3537	10µmol/L
Propranolol hydrochloride (Pro)	Sigma-Aldrich	P3500000	10µmol/L
Bisoprolol fumarate (Bis)	Sigma-Aldrich	Y0000812	10µmol/L

1.3.Murine ventricular cardiac slice culture

Animal handling, breeding, and sacrifice were performed under approval of regulations of the regional government of Upper Bavaria (Gz.: 55.2-1-54-2532-139-2014). Mice were purchased from The Jackson Laboratory. 6-8 weeks old, age and sex-matched littermates of *Jup* (junctional plakoglobin, gene coding for plakoglobin protein) WT and KO mice were used for experiments. After sacrifice by cervical dislocation, mice hearts were immediately removed, placed in pre-cooled oxygenated cardiac slicing buffer and embedded in low melt agarose. After hardening, 200 µm thick ventricular cardiac slices were cut with a LeicaVT1200S vibratome (Leica Biosystems). For dissociation assays, the slices were washed gently with HBSS and transferred to pre-warmed cardiac slices medium, containing DMEM 1:1 F12 nutrient mixture (life technologies, #21331020), 10% foetal bovine serum, 1% minimum essential medium nonessential amino acids (gibco, #11140), 2 mM L-glutamine, 10 U/L penicillin and 10 µg/mL streptomycin in 24-well plates and incubated with indicated mediators for 60 minutes at 37°C, 5% CO_2.

1.4.Immunostaining

The following primary antibodies were used for immunostaining: N-CAD (BD Transduction, #610921) and DSG2 (Progen, #610121)). Primary antibody incubations were performed at 4°C overnight in a wet chamber. After washing, species-matched, fluorophore-coupled secondary antibodies (anti-mouse Alexa-488 1:100 (Dianova #115-545-003) and anti-rabbit Cy5 1:600 (Dianova, 111-175-144)) were applied for 60 minutes at room temperature. In the last 10 minutes of secondary antibody incubation, DAPI (Roche, #10236276001) was added. Coverslips were mounted on microscope slides with NPG (Sigma-Aldrich, #P3130). All washing steps and reagent dilutions were performed in PBS.

1.5.Histological analysis.

Hearts were dissected and fixed in 4% PFA, dehydrated, and embedded in paraffin.

Global heart morphology was determined from transversal 10 µm deparaffinized sections stained with hematoxylin and eosin (H&E). Fibrosis was detected with picrosirius red stain. Samples were analyzed in a Keyence Biozero BZ-800 microscope. Hearts from 6 different animals were quantified for each experimental group.

For immunohistochemical analyses, hearts were dissected and fixed in 4% PFA, dehydrated, and embedded in paraffin. Sections (10 µm) were cut, mounted, deparaffinized in xylene and rehydrated through an ethanol series. After a 10 minutes treatment at 95°C in a citric acid based antigen unmasking solution (Vector Laboratories, H-3300), sections were incubated in 0.3% H_2O_2 in methanol for 10 minutes. Sections were then permeabilized for 10 minutes using 0.2% Nonidet, rinsed, blocked with M.O.M® Mouse IgG (FMK-2201) for 60 minutes and then incubated overnight with mouse anti-plakoglobin (#61005, Progen). After washing, sections were incubated with the diluted biotinylated secondary antibody for 30 minutes, rinsed and incubated with VECTASTAIN Elite ABC reagent (PK-6100, Vector Laboratories) for 30 minutes. After final washing, sections were developed in a peroxidase substrate solution (ImmPACT® DAB, SK-4105, Vector laboratories), and mounted. Samples were analyzed in a Keyence Biozero BZ-800 microscope. The hearts from 6 different animals were quantified for each experimental group.

1.6. Dissociation Assays in HL-1 cells.

Under basal conditions: medium was supplemented with 1.8 mmol/L Ca^{2+}. After respective treatments (60 minutes for mediators, inhibitors were added 30 minutes in advance) cells were washed with HBSS, treated with Liberase-DH (Sigma-Aldrich, #5401054001) and Dispase II (Sigma-Aldrich, #D4693) and incubated at 37°C until the cell monolayer detached from the wells. The enzyme mix was carefully removed from the wells and replaced by HBSS. Mechanical stress was applied by pipetting up and down four times with an electrical pipette.

When dissociation assays were performed after siRNA treatment, mechanical stress was applied to the cells by shaking at 1250 rpm for 5 minutes on an orbital shaker. MTT (Sigma, #M5655) was applied to the wells to check cell viability. Images of the wells were taken to count the number of fragments using ImageJ software.

Under Ca^{2+}-depleted conditions: Cells were detached from the wells with Liberase-DH and Dispase II, and supplemented with Claycomb medium along with the mediators for 60 minutes, inhibitors were added 30 minutes in advance. After incubation, the medium was carefully removed and replaced by fully supplemented, EGTA-containing Claycomb medium along with the mediators for another 90 minutes. Finally, the cell monolayers were mechanically agitated as explained above and the resulting fragments were imaged and counted using ImageJ software.

1.7.Dissociation Assays in murine cardiac slices.

When performing dissociation assays with murine cardiac slice cultures, slices were treated with EGTA or mediators as described above, washed once with HBSS and Liberase-DH was added together with Dispase II for 25 minutes at 37°C. 0.75 volumes of HBSS was added to each well, and mechanical stress was applied using an electrical pipette by pipetting up and down four times. After a settling time of 5 minutes, medium containing cell debris and detached cells were filtered using 70 µm nylon membrane, and the number of dissociated cardiomyocytes was counted with an inverted microscope (Axio, Carl Zeiss, Oberkochen, Germany) and taken as an indirect measurement for intercellular adhesion. To control variations due to different slice size or location in the ventricle, consecutive slices for control and treatment were used, respectively, and the result of a slice was normalized to the respective control slice.

1.8.Western Blot analyses

The following antibodies were used as primary antibodies: anti-phospho ERK1/2 ((E-4) Santa Cruz, #sc-7383), anti-phospho p38MAPK ((D3F9) Cell Signaling, #4511), anti-ERK1/2 (Cell Signaling, #9102), anti-p38MAPK (Cell Signaling, #9211), anti-phospho PG, anti-PG ((PG5.1) Progen #61005), anti-DP1+2 ((2.15), Progen, #61003) and anti-α-tubulin ((DM1A), abcam, #7291). Blots were incubated with primary antibodies overnight at 4°C on a rocking platform. Horseradish peroxidase-conjugated were used as secondary antibodies (Dianova, #111-035-045 or #115-035-068) and applied for 60 minutes at room temperature. Apart from anti-PG and anti-α-tubulin, primary antibodies were used at 1:1000 dilutions. Anti-pPG was used in a 1:20 dilution and anti α-tubulin was used at 1:4000. Secondary antibodies were diluted 1:10000. All antibodies were diluted in TBS-Tween. Quantification was performed using ImageJ software.

1.9.siRNA knockdown

Cells were transfected with siRNA 24h post-seeding. Therefore, the transfection reagent, containing OptiMEM (Thermo Scientific, #31985070), RNAiMAX (Thermo Scientific #13778150) and the ON-Target siRNA (Dharmacon, siNT #D-001810-10, siDsg2 #L-042514-01, siN-Cad #L-040206-00, siJup #L-040316-01, siDsp #L-040653-01) was added according to manufacturer's protocol.

2. Supplemental figures

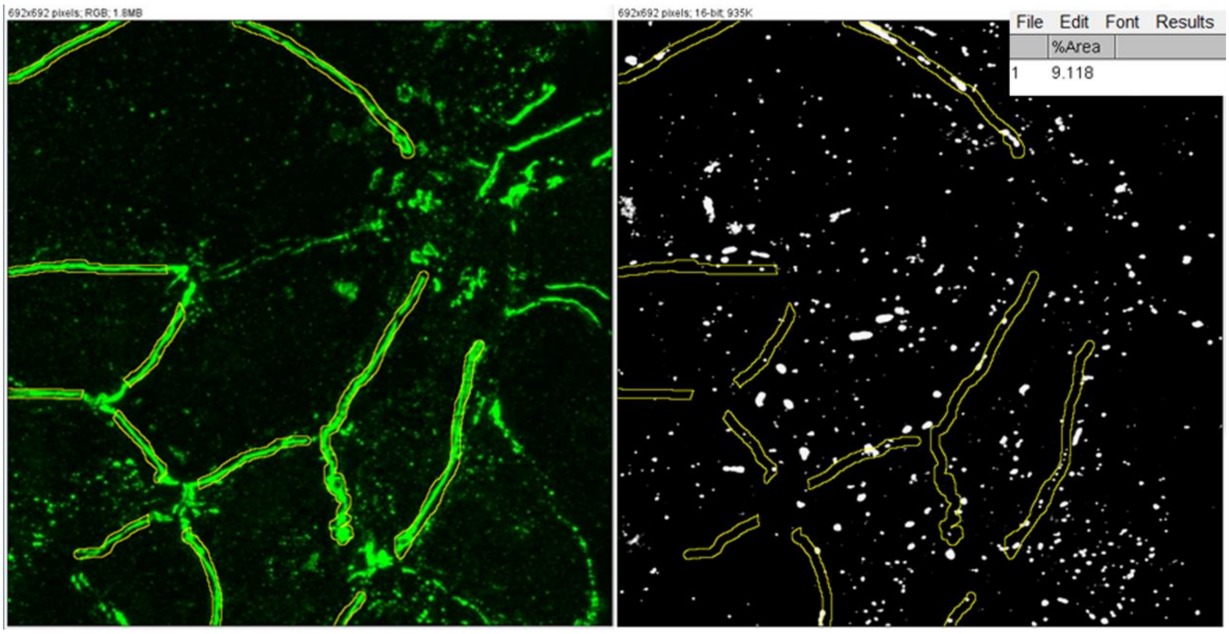

Supplemental Figure 1. Immunofluorescence quantification

Exemplary quantification of N-CAD/DSG2 colocalization. Left panel shows N-CAD staining in a control sample with the ROI (yellow lines); right panel shows corresponding DSG2 staining with the ROI. Table (top right) shows pixels not equal to zero, which corresponds to the amount of colocalization calculated by ImageJ

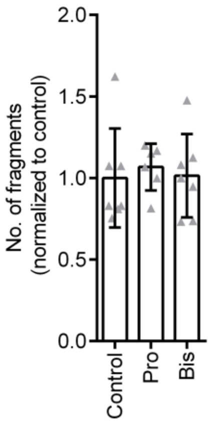

Supplemental Figure 2. Effect of β-adrenergic receptor blockers on cardiomyocyte adhesion

Dissociation assays in HL-1 cardiomyocytes treated with propranolol (Pro) and bisoprolol (Bis) for 60 minutes. One-Way ANOVA with Holm-Sidak correction, n=7.

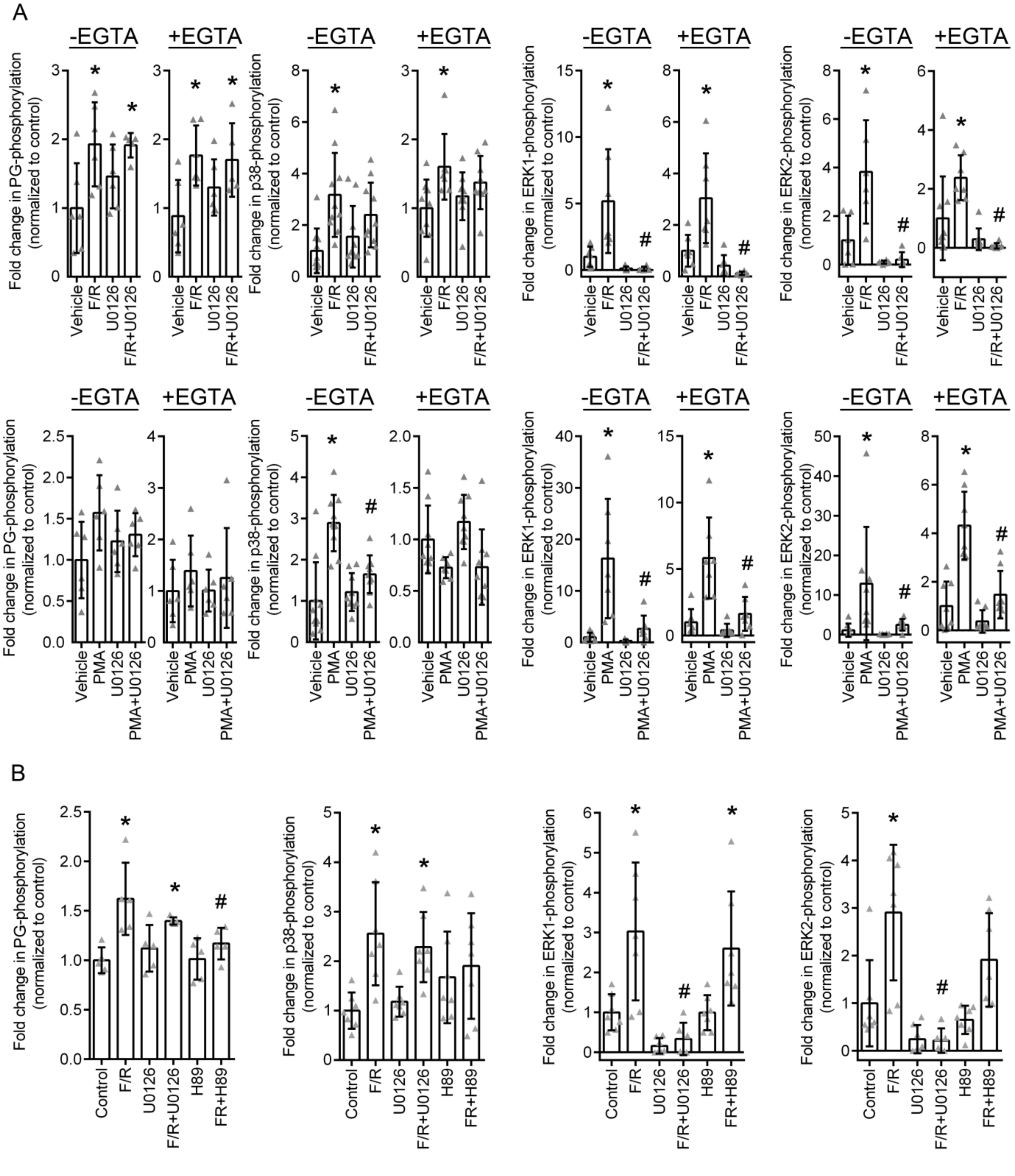

Supplemental Figure 3. Western blot quantification following F/R or PMA with and without U0126 treatment under basal and Ca^{2+}-depleted conditions

A: Quantification for Western blots of HL-1 cells treated with F/R, Iso, and PMA with and without U0126 under basal and Ca^{2+}-depleted conditions, as was done in Figure 3B. Fold

changes in phosphorylation normalized to the respective controls conditions were plotted. * and # indicate p≤0.05, One-Way ANOVA with Bonferroni-correction, * indicate significance as compared to the control, # indicate significance as compared to the respective U0126-untreated condition, n=6-9. **B**: Quantification for Western blots of HL-1 cells treated with F/R, with and without U0126 or H89 under basal conditions, as was done in Figure 3D. Fold changes in phosphorylation normalized to the respective control conditions were plotted. * and # indicate p≤0.05, One-Way ANOVA with Bonferroni-correction, * indicate significance as compared to the control, # indicate significance as compared to F/R, n=6

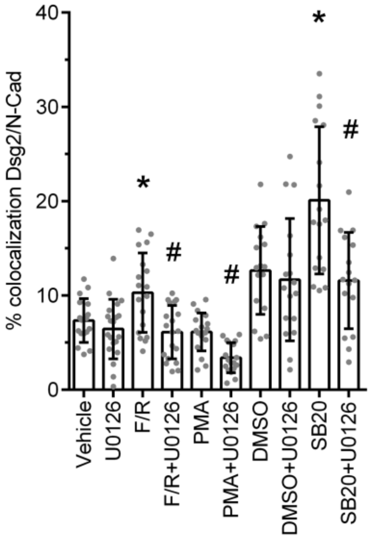

Supplemental Figure 4. Colocalisation analysis of DSG2 and N-CAD

Quantification of the colocalization of DSG2 and N-CAD under basal conditions. * and # indicate p≤0.05, One-Way ANOVA with Bonferroni-correction, * indicates significance as compared to the respective control, # indicate significance as compared to the respective U0126-untreated condition. Each data point represents one individual area in one slide. In total n=6 was performed in different cell passages.
